# The "Water Duties" Personnel of the Territorial Army

**Published:** 1908-10-31

**Authors:** 


### The Royal Army Medical Corps Section.

### THE "WATER DUTIES" PERSONNEL OF THE TERRITORIAL ARMY.

In an article on camp sanitation in The Hospital for August 8 we emphasised the fact that, in the main, disease is preventable, and that in camp life the factors that ensure the good health of the troops are: (1) purity of water; (2) good and well-cooked food; and (3) the proper disposal of excreta, and camp refuse. Of these three, the first is, perhaps, of the greatest importance, so many diseases being water-borne. In standing camps, or in camps established on selected sites for a period of annual training, there is no special difficulty in ensuring a safe and abundant water supply and, by ordinary precautions, protecting it from contamination. It is when troops are on active service or on the march, be it for field days or manœuvres, that danger arises from the water used for drinking purposes. Hence, if health is to be preserved, the same care is necessary under these conditions as in camp, and a system of sanitation that does not recognise this fact and provide for the safety of the troops wherever they may be placed is of no value, as it will not be successful.

Recognising fully the importance of this principle our military authorities have made provision in the medical service of the new Territorial Army for the attachment to units, just as in the Regular Army, of a *personnel* for "water duties." This *personnel* will be in addition to that distributed to the combatant units for general and medical sanitary work, and the non-commissioned officers and men composing it will belong to the R.A.M.C. (T.). They will be enlisted for this special branch of work and they will be trained accordingly. The units of every branch of the service—Cavalry, Artillery, Engineers, Infantry, and Army Service Corps—will possess this water-duty *personnel* in varying proportions, the actual numbers allotted to each unit being given in a table on page 10 of the "Field Service Manual," for 1908. Collectively, the numbers required for a division total up to about 20 corporals and 100 men. It must be understood that these men for water-duty are only attached to combatant units when the latter are mobilised or when they go into camp for annual training. Whilst so attached the men will, subject to the regimental commanding officers, be under the orders of the regimental medical officers, and they will be employed only on water purification and disinfection duties; but they may also be called upon by the regimental medical officers to give general assistance in the field.

The enlisting and training of a special body of men for water-duty necessitates careful consideration as to the best way of carrying it out. Different suggestions have been put forward, but it seems clear from the recent Army Order, 219, paragraph 17, of September 1, that it has been decided to raise the men as distinct bodies. The regulations says: "These men will be raised in or near centres where field ambulances exist in sufficient numbers to meet the requirements of the divisions, and, except when mobilised with their combatant units, will be attached to the field ambulances and be trained by them with the assistance of the regimental officers." This plan has many advantages over the alternative of having the men enlisted by the individual units themselves. Were this latter course to be adopted it would give rise to difficulties in their training and the expense would be greater. On the other hand, the proposed arrangement will facilitate training and ensure uniformity of instruction, as the centres chosen are those of the field ambulances, at whose headquarters there are stationed the divisional schools of instruction; thus enabling these schools to be available for teaching purposes and allowing, too, of the divisional sanitary officer assisting, if he desired, in the instruction given. The only difficulty connected with this scheme is the apportioning of the expense of clothing these water-duty men amongst the various county associations for whom they are being trained, but this is a point of administrative detail that should be easily settled.

The training of these water-duty men will vary with the period of their service. During their *recruit's course* they will make the necessary attendances at the headquarters of the field ambulance to which they are attached to learn what is known as "setting up" and infantry drill. This is of an elementary nature and extends only to a knowledge of saluting, marching, and the simpler formations. This over, they take up their training at the school of instruction in the special subject of sanitation. The course for this extends over eight days, on each of which there is either a lecture or some practical instruction. The syllabus of instruction embraces the following subjects: (1) The object of sanitation and its importance to the army; (2) causation of disease, with special reference to preventable and water-borne diseases; (3) the common sources of water and its impurities; (4) methods of clarifying, filtering, and sterilising water; (5) the army filter-cart, its construction and management, with practical instruction in its use; (6) disinfection; (7) camp sanitation, with the disposal of sewage and refuge; (8) personal hygiene in barracks and on the march. At the close of the course a qualifying examination is held, and when this is passed successfully the first period of training comes to an end. The after recruit's training of water-duty men consists in their carrying through every year one of the three following conditions: (1) making the necessary number of attendances, 10, at the headquarters of the field ambulance to which they are attached; (2) attending a course of sanitation at a R.A.M.C. (T.) school of instruction; (3) training in camp with the unit to which they are attached for duty.

It will be remembered that the director-general, when making last year his first announcement on the proposed medical service for the Territorial

Army, pointed out that his scheme introduced three new features that had no existence in the old volunteer medical service, namely: the establishment (1) of administrative medical officers for each division; (2) of general hospitals; and (3) of a sanitary branch. Good progress has been made with all these three improvements, and the sanitary branch as now constituted is made up of 120 sanitary officers (composed of 20 lieutenant-colonels, 40 majors, and 60 captains), of two sanitary companies, each consisting of 5 officers and 100 men of other ranks, and of the above described water-duty *personnel* attached to individual units. Such a sanitary branch should be a valuable agent in preserving the health of the troops, but the provision of a special body of men for any particular duty is apt to engender the idea that others are entirely freed from anything to do with it. This does not hold good with sanitation, which can only be successful in the every-day life of the army and in the field if individual units recognise their duty and responsibility in the matter. To bring this home to them and to place within each unit a valuable organisation for this work the regulations provide that in every regiment one non-commissioned officer and eight men shall be selected for sanitary duties, their training to be in the hands of the regimental medical officer. No doubt this sanitary teaching will throw on this officer additional work, but it enables him to be of incalculable service to his battalion. In fact we consider that under the new Territorial medical service the status of the regimental medical officer is greatly improved. He is now placed in a position of greater responsibility and usefulness than before, and we propose in another article to dwell further on this point.

